# Professor Ogobara K. Doumbo (1956–June 9, 2018)

**DOI:** 10.4269/ajtmh.18-1956

**Published:** 2018-10-22

**Authors:** Abdoulaye Djimdé, Louis H. Miller, Christopher V. Plowe

**Affiliations:** 1 Malaria Research and Training Center, University of Sciences, Techniques and Technologies of Bamako, Bamako, Mali;; 2 Laboratory of Malaria and Vector Research, National Institute of Allergy and Infectious Diseases, Bethesda, Maryland;; 3 Duke Global Health Institute, Duke University, Durham, North Carolina

The tropical medicine and global health community has lost one of its heroes. Professor Ogobara (“Ogo”) K. Doumbo died on June 9, 2018, after a brief illness. Professor Doumbo’s life and accomplishments have been described in an obituary in *The Lancet*^1^ and during his lifetime in profiles in *The Lancet*^2^ and *Science*.^3^ This is a personal remembrance from three of his friends and colleagues.

**Abdoulaye Djimdé:** Professor Ogobara Doumbo’s contributions to science, health care, and public health are numerous, spanning from health systems to humanitarian work, higher education, and basic and applied research. The Malaria Research and Training Center in Bamako, Mali, which he cofounded and directed, is one of the premier research centers in Africa. The center produced a number of landmark studies on malaria and other parasitic diseases and trained hundreds of young scientists from Africa, the Americas, Asia, and Europe. Beyond his achievements as a scientist and trainer, Professor Doumbo was a superb leader and mentor. His caring, loving, and personal touch in his interactions with each of his coworkers and students created a family atmosphere in the workplace. For his coworkers, Professor Doumbo was not just the Boss but also a mentor, a big brother for some, a father figure, or a grandfather figure for others. Every moment with him was an opportunity for learning, not just about science but about life. He will be greatly missed by all of his colleagues, his students, and his family, which included 10 children and six grandchildren.

**Louis H. Miller:** I first got to know Ogo in 1988, when I worked with him and with his colleague, entomologist Yeya Touré, to develop a malaria research program in Mali supported by the Rockefeller Foundation and the World Health Organization’s Special Programme for Research and Training in Tropical Diseases. Under the socialist government that ruled Mali in the 1960s, one child from each family was given the opportunity to receive an education. Ogo was chosen by his family to leave his village in the Dogon Country to be educated. He eventually completed medical school at the University of Bamako, where he met French parasitologist Philippe Ranque, who sponsored Ogo to earn a PhD in Marseilles. But before starting his PhD, like all medical graduates, Ogo had to spend 2 years doing clinical service. He was sent to Banamba and then to Sélingué in the south of Mali, where he decided that teaching public health required evidence that he could help the population. He did so by saving the lives of women with difficult pregnancies by Caesarean section. After this, he felt the population would listen to him. Throughout his life, he was original in his approach to public health. We would go in the field often, where I learned so much about Ogo. When we went to Sélingué, he showed me a hospital that he convinced a wealthy German woman to build. Quite an accomplishment for a young doctor from the Dogon Country. He also showed me a movie which he produced with Philippe Ranque to tell the story of dracunculiasis and how community members could eliminate the disease. It was voiced over by Catherine Deneuve.

Following his PhD training, Ogo progressively built a team of talented Malian scientists to develop research to control and eliminate malaria. Among his early protégés were Mahamadou Thera, who ran the vaccine program; Alassane Dicko, who did drug trials; and Abdoulaye Djimdé, who led research on drug resistance. Djimdé performed important studies on the genetic basis of chloroquine resistance, published in the *New England Journal of Medicine,*^4^ and later became a Howard Hughes Medical Institute International Research Scholar. Ogo and his team went back to the Dogon Country to start a field research site in Bandiagara, where they saw many children dying of cerebral malaria. Ogo immediately developed a rapport with the community to set up a referral system for specialized care for severe malaria. Again, he was saving lives through a sensible approach to the local situation. This visit led to studies by Ogo and National Institute of Allergy and Infectious Diseases malariologist Tom Wellems that found that hemoglobin C was protecting Dogon children against severe malaria.^5^

Just last year Ogo offered me the opportunity to work on *Plasmodium vivax* in Duffy-negative children in Bandiagara.^6^ There was no clinical evidence of infection, and the blood films were read as negative on 2% of children who were polymerase chain reaction-positive for *P. vivax.*^6^ The only clinical problem was anemia that may have been from the *P. vivax*. We planned to continue the studies for another few years, but now, because of Ogo’s sudden death, I will be working with his colleague, Professor Thera.

**Christopher V. Plowe:** Ogobara Doumbo was my friend, collaborator, and mentor—I often referred to him as my big brother. When I met him in 1992, I was a fellow in Tom Wellems’ lab at National Institutes of Health, a clinician with aspirations to do field research, struggling to find my way in a basic science lab. I was working on molecular markers of drug-resistant malaria, and Ogo immediately saw the potential of using these markers as tools for surveillance. On our first trip to Mali, Ogo assigned a recent PharmD graduate to work with Tom and me as we designed the first nested PCR for resistance mutations in the Malaria Research and Training Center lab in Bamako. This student, Abdoulaye Djimdé, became a leading member of the “F1” generation of Ogo’s trainees who have become internationally recognized as scientific leaders in Africa.

As Djimdé noted, Ogo’s contributions to science and global health were protean, but if I had to name the greatest impact of his life’s work, it would have to be the multiplier effect of his unique ability to identify, groom, mentor, and support research trainees who had the talent and commitment to make important contributions to malaria research. These trainees—by now, at least three generations of them—have not only become leaders themselves but they have played forward that same spirit of generosity and commitment to improving the health of the people of Mali and the world—especially of people living in the remote, rural villages who suffer the most from tropical parasites such as malaria and schistosomiasis.

Ogo came from this world, and never forgot it. As a young boy, he spent half of each year in a village school, and the other half roaming the Dogon Plain as the chief of a crew of boys who herded goats (later graduating to cows), living outdoors, and fending for themselves for months at a time. He was 16 years old the first time that he saw the interior of an automobile.

When he returned to the Dogon Country in the mid-1990s with Tom, Djimdé, and me to setup a field site to study severe malaria, Ogo understood that if we wanted to know why we were finding so few cases of cerebral malaria in the Bandiagara hospital, we needed to talk with the local traditional healers. Through his patient and respectful investigation, we learned that in the Dogon Country, hundreds of children each month were taken to traditional healers for “Wabu,” a spiritual disease manifested by fever and coma that occurred when a mystical bird flew over a child and cried out at the same moment that the child cried. Wabu was treated with herbal remedies and incantations.

Earning the trust of the healers, Ogo probed further and learned that they kept careful pictographic records of the outcomes of Wabu—which had a case fatality rate in Bandiagara of about 50%, the textbook mortality rate for untreated severe malaria. Ogo was then able to negotiate an agreement between the Traditional Healers Association of Bandiagara and the hospital staff that healers would refer cases of Wabu to the hospital for antimalarial treatment, which would be administered by doctors and nurses trained by Ogo and his research team. After they recovered, children were referred back to the healers, who were credited for making the right diagnosis and referral. Fatality rates for severe malaria decreased more than 10-fold, and the resulting good will in the community contributed to the success of a large malaria research program in Bandiagara.

This example of building trust and respect between practitioners of traditional and modern medicine stayed with me years later when I began a project in another country, where I heard traditional healers referred to dismissively as “quacks.” At that moment, and in many others since, I have found myself asking, “What would Ogo have to say about this?” I can imagine his dignified, wise voice gently offering sound counsel to do the right thing based on ethics, respect, justice, fairness, and integrity, values that he personified and transmitted to generations of trainees, who will continue to propagate them for decades to come.

**Figure:**
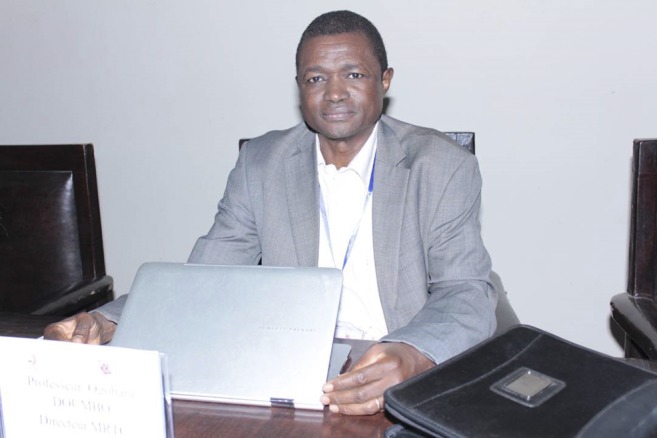
**Professor Ogobara K. Doumbo (1956–June 9, 2018)**

## References

[1] Green A, 2018 Ogobara Doumbo. Lancet 392: 116.

[2] Pincock S, 2008 Ogobara Doumbo: building capacity for malaria research in Africa. Lancet 372: 1537. 18984177 10.1016/S0140-6736(08)61640-2

[3] Taylor DA, 2011 Profile: Ogobara Doumbo. Mali researcher shows how to reverse brain drain. Science 332: 1498–1499. 21700851 10.1126/science.332.6037.1498

[4] Djimdé A 2001 A molecular marker for chloroquine-resistant falciparum malaria. N Engl J Med 344: 257–263. 11172152 10.1056/NEJM200101253440403

[5] Agarwal A 2000 Hemoglobin C associated with protection from severe malaria in the Dogon of Mali, a West African population with a low prevalence of hemoglobin S. Blood 96: 2358–2363. 11001883

[6] Niangaly A 2017 *Plasmodium vivax* infections over 3 years in duffy blood group negative malians in Bandiagara, Mali. Am J Trop Med Hyg 97: 744–752. 28749772 10.4269/ajtmh.17-0254 PMC5590610

